# Processing Uncertain RFID Data in Traceability Supply Chains

**DOI:** 10.1155/2014/535690

**Published:** 2014-03-10

**Authors:** Dong Xie, Jie Xiao, Guangjun Guo, Tong Jiang

**Affiliations:** ^1^Department of Information Science and Engineering, Hunan Institute of Humanities, Science and Technology, Loudi 417000, China; ^2^Department of Information Science and Engineering, Hunan First Normal University, Changsha, 410205, China; ^3^Department of Electronics and Information Engineering, Loudi Vocational and Technical College, Loudi 417000, China

## Abstract

Radio Frequency Identification (RFID) is widely used to track and trace objects in traceability supply chains. However, massive uncertain data produced by RFID readers are not effective and efficient to be used in RFID application systems. Following the analysis of key features of RFID objects, this paper proposes a new framework for effectively and efficiently processing uncertain RFID data, and supporting a variety of queries for tracking and tracing RFID objects. We adjust different smoothing windows according to different rates of uncertain data, employ different strategies to process uncertain readings, and distinguish ghost, missing, and incomplete data according to their apparent positions. We propose a comprehensive data model which is suitable for different application scenarios. In addition, a path coding scheme is proposed to significantly compress massive data by aggregating the path sequence, the position, and the time intervals. The scheme is suitable for cyclic or long paths. Moreover, we further propose a processing algorithm for group and independent objects. Experimental evaluations show that our approach is effective and efficient in terms of the compression and traceability queries.

## 1. Introduction

RFID technology [1] is a flexible and relatively low-cost solution for tagging and wireless identification in a large number of business applications. Every tagged RFID object has a unique identifier, which can be automatically identified and collected to generate massive data by RFID readers. In recent years, the technology has become increasingly popular in the supply chain management (SCM) [1]. In supply chains, RFID applications cross multienterprises such as manufacturing, dealer, wholesaler, retailers, and customers and obtain EPC-tagged RFID products including their specifications and positions. RFID applications are available for users to track and trace historical trajectories and concrete positions of RFID objects over the Internet according to their related position information denoted as data lineages [2].

Although most RFID data are precise, RFID devices are intrinsically sensitive to environmental factors. The uncertainties inevitably exist in RFID data and significantly complicate tasks of determining objects' positions and containment relationships [2]. However, most works focus on certain RFID data processing rather than uncertain RFID data [3, 4]. As a result, RFID applications still face significant challenges for managing uncertain RFID data [2].

First, as RFID applications focus on positions of RFID objects at different times rather than information of RFID readers, traditional data models such as in [3, 4] often employ a triplet (*EPC*,* reader_id*, and* timestamp*) to store reading data. In recent years, some works [5–21] in terms of uncertain data have been studied in other application environments such as data integrations. User queries are redefined to obtain precise query answers with a confidence above a probabilistic threshold over uncertain data by modifying query predicates. Moreover, user queries are rewritten as “consistent” retrieval for resolving inconsistencies and probabilities.

However, these approaches do not consider features of RFID applications such as temporal and spatial relationships of objects. As a result, path-oriented queries cannot be directly processed on uncertain RFID data without preprocessing in complex cases of RFID environments. In addition, path-oriented queries need to obtain all information at all logistic nodes, which may be inefficient over massive objects. As a result, these methods are difficult to store and process uncertain data for expressing RFID objects' movements. It is necessary to model RFID data under a unified model for preprocessing uncertain RFID data.

Second, the smoothing window size significantly affects the rate of different types of uncertain data such as missing, inconsistent, and ghost data [22–24]. But existing works still are not effective to determine a variable window for all possible RFID data streams [23]. Moreover, inference rules need to be developed for processing different types of uncertain data. However, existing works do not comprehensively consider all types of data such as inconsistent and incomplete data.

Third, most path-oriented queries are inefficient because the queries perform multiself-joining of the table involving many related positions under traditional data model [3, 4]. Some works [25–27] compress certain RFID data, and path-oriented queries can efficiently obtain historical path information of object movements. However, the methods are ineffective because object moving may be a cyclic or long path in supply chains, and this further complicates path-oriented queries.

Therefore, an important challenge lies on how to develop inference rules for effectively processing uncertain data captured by readers according to adjusted smoothing windows. Another challenge is how to compress massive data efficiently for processing uncertain data under a comprehensive data model.

In this paper, we propose a framework for processing uncertain RFID data. According to key features of RFID applications, we present a comprehensive data model for storing uncertain RFID data and employ a strategy to adjust sizes of smoothing windows for capturing suitable rates of different uncertain RFID readings. We further develop inference rules for different types of uncertain data and propose a path coding scheme called path (sequence) for compressing massive data by efficiently aggregating the path sequence, the time interval, and the position. The coding scheme also resolves the object moving problems with cyclic or long path. Specially, we consider that the query answers do not need precise information such as concrete reading timestamps and transferring positions. The main contributions of our work are summarized as follows.

- We summarize key features and classify entities of RFID applications. We propose a framework for preprocessing raw uncertain RFID data including ghost, missing, redundant, inconsistent, and incomplete data. Also, we employ a strategy to determine suitable rates of different uncertain RFID readings by adjusting the size of smoothing windows. Inference rules of different uncertain RFID data are developed. Our framework can distinguish ghost, missing, incomplete (fake), and incomplete (stolen object) readings according to their apparent positions in supply chains.
- We propose a comprehensive data model which is suitable for different application scenarios. It considers most properties of objects captured by RFID readers. According to the preprocessing framework, deployment, reading, and outline data are stored or generated in the deployment, production, warehouse, transfer, and retail stages, respectively.
- We propose a path coding scheme to significantly compress massive data by aggregating the path sequence, the position, and the time interval. The scheme also expresses object movements with cyclic or long paths. We further comprehensively consider objects with group and individual movements and propose a preprocessing method for raw data. First, it executes the aggregating algorithm for compressing different granularity data; it then executes the graph algorithm to build the directed graph with respect to paths of object movements; finally, it completes the preprocessing of uncertain data.
- We conduct extensive experimental evaluations for our model and the proposed algorithms. Stay records are generated instead of raw RFID data to reflect real environments. We formulate seven tracking, tracing, and aggregate queries to further test the affection in terms of the compression, the aggregate levels for the position and the time interval, the data size, the path length, and the number of grouping. Experiment evaluations show that our methods are effective and efficient.

The rest of the paper is organized as follows.  Section 2 presents the related works.  Section 3 describes the preliminary and the system framework of RFID applications.  Section 4 presents inferring rules for uncertain data.  Section 5 presents the data model. The path coding scheme is discussed in Section 6.  Section 7 introduces a variety of queries.  Section 8 reports the experimental and performance results. Finally, Section 9 offers some concluding remarks.

## 2. Related Works

It is difficult to model RFID data by using the traditional ER model because RFID data are temporal. In recent years, some data models have been proposed to process RFID data. However, few models have been designed to effectively model uncertain RFID data and efficiently process various uncertain data. Dynamic Relationship ER Model (DRER) [3] discusses two types of historical data in the scenario of fixed readers: event-based and state-based. The model is further classified into a set of basic scenarios to develop constructs for modeling each scenario according to fundamental characteristics of RFID applications [4].

Most path-oriented queries use data lineages to trace movement histories over uncertain data, so user queries should be redefined for cleaning data from users' point of view and for uncertain data from the system's point of view. Some works (e.g., probabilistic range queries [5], probabilistic nearest neighbor queries [5], probabilistic similarity join [6], probabilistic ranked queries [7], probabilistic reverse skyline queries [8], and probabilistic skyline queries [9]) in recent years in terms of uncertain data have been studied. These redefined query types modify query predicates to obtain precise answers with a confidence above a probabilistic threshold over uncertain data. Also, effective pruning methods have been proposed to avoid accessing all objects in the database for reducing the search cost.

Most previous works focus on resolving inconsistencies and probabilities. Inconsistencies have two major aspects: database repair and* consistent query answer* (CQA). The existing repair models can be classified into five types: (a) the minimal distance-based repair to the inconsistent database generates the maximum consistent subset of the inconsistent database [10]; (b) the priority-based repair prefers more reliable data sources according to different reliabilities of conflicting data sources [11]; (c) the attribute-based repair minimizes attribute modifications [12]; (d) the cardinality-based repair semantics minimizes the number of whole tuples [13]; and (e) the null-based repair stores null values for representing data consistencies [14]. However, existing CQA methods, such as query rewriting [15], conflict graph [16], and logic program [17], only retrieve “consistent” answers over inconsistent databases with respect to a set of integrity constraints but do not repair the inconsistent database. An aggregation query returns a different answer in every repair, so it has no consistent answer for CQA. Under the range semantics [18], computing a minimum and maximum value of a numerical attribute of a relation can be expressed as first-order queries. The method in [19] can return the minimal interval, which contains the set of the values of the aggregate function obtained in some repair.

All the works mentioned above cannot be extended to uncertain RFID data with the possible worlds' semantics, which may appear together in the real world as all the possible combinations of data instances [20]. Probabilistic consistent query answering (PCQA) [20] utilizes all-possible-repairs semantics and studies all possible repairs and possible worlds for CQA in the context of inconsistent probabilistic databases. The CQA satisfies the query predicates with high consistency confidence above a probabilistic threshold. A framework is proposed to process uncertain data over Internet of Things (IoT) [21], and the method represents each IoT object using 7 tuples of the form 〈*EPC, instance_id, attribute, value, time, probability, and reliability*〉. The method provides a neat representation of IoT data in the form of probabilistic streaming data. However, these two works did not consider key features of RFID applications, so they cannot be directly used in complex cases of an RFID environment. For example, an object has 2 sets of inconsistent positions (e.g.,* V2* and *V*2′ are in a wholesaler and* V3* and *V*3′ are in a dealer) at 2 different logistics node (e.g., a wholesaler and a dealer). Since path-oriented queries need to obtain all information at all logistic nodes rather than all possible worlds, PCQA is unsuitable for path-oriented queries. Moreover, this situation might cause extra exponential overload for path-oriented queries over massive objects.

Situations of low-cost, low-power hardware and wireless communications lead to frequently dropped readings with faulty individual readers. A smoothing window significantly affects the rate of redundant, inconsistent, missing, and ghost data [22–24]. A data cleaning technique for sensor stream is proposed [22], but it is difficult to obtain rougher position granularities because the size of the smoothing window is not suitable. SMURF [23] automatically and continuously determines the smoothing window size by sampling based on observed readings of the detection system, but it is not effective to determine a variable window for all possible RFID data streams.

To further process uncertain data, inference rules need to be developed for processing uncertain data. Spire system [28] employed a time-varying graph model to efficiently estimate the most likely position and containment for each object over raw RFID streams. A scalable, distributed stream processing system for RFID tracking and monitoring [29] proposes novel inference techniques that provide accurate estimates of object positions and containment relationships in noisy, dynamic environments. A probabilistic data stream system called Claro uses continuous random variables for processing uncertain data streams [30]. Claro develops evaluation techniques for relational operators based on a flexible mixed-type data model by exploring a convex combination of Gaussian distributions. However, these works do not comprehensively consider all types of data such as inconsistent and incomplete data. Inference rules need to be developed for processing uncertain data.

Path-oriented queries require performing multiself-joining of the table involving many related positions. Gonzalez et al. [25] focus on group of objects and use compression to preserve object transition relationships for reducing the join cost of processing path selection queries. Their work in [26] extends the idea to account for a more realistic object movement model and further develops a gateway-based movement graph model as a compact representation of RFID data sets. Lee and Chung [27] focus on individual objects and propose a movement path of an EPC-tagged object, which is coded as the position of readers and the order of positions denoted a series of unique prime number pairs. However, their approaches do not code longer paths, which may lead to the overflow of the prime numbers. The method only supports the product of the first 8 and 15 prime numbers for 32 bits and 64 bits, respectively. In addition, since an object may have the same position with different time intervals, the method is not applicable to express cyclic paths. Ng [31] uses finite continued fraction (rational numbers) to represent respective positions and their orders, whose paths may be long and cyclic. However, rational numbers notably increase the volume of data and inefficient queries arise. These methods can provide compression and path-dependent aggregations, but they have not adequately considered uncertainties of RFID data.

## 3. Preliminary

### 3.1. RFID-Based Supply Chains

**Figure 1:**
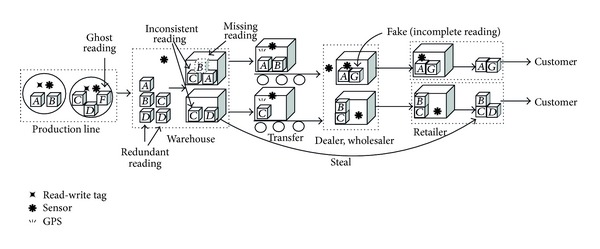
A RFID-based supply chain.

In the whole process of the RFID-based supply chain as shown in Figure 1, all readings from RFID readers are automatically captured. First, each product item is tagged with an electronic product code (EPC) in the production line and related product specifications are written into tags. Second, products with tags are packed into cases in supplier warehouses. The EPC-tagged cases are then packed onto pallets at the supplier warehouses, where the EPC tags of both the cases and the containing pallets are scanned by RFID readers. After that, pallets are loaded onto trucks, which then depart to dealers. At zones of retail stores, all pallets are unloaded from the trucks, and all cases are unpacked from the pallets. Eventually, product items are purchased by consumers.

Specially, sensors may be attached on EPC tags of objects or positions. In most periods of the supply chain, sensors may measure environmental parameters of RFID objects such as temperature and humidity. These parameters are helpful to protect objects. For example, food may spoil when their temperatures exceed a certain value. In addition, GPS sensors might be attached on EPC-tagged trucks for measuring their positions in the transportational process.

### 3.2. Key Features of RFID Applications

Despite different types of RFID applications, their general features are the same. Information of EPC-tagged objects is captured at certain time by certain RFID readers. We summarize key features of RFID applications as follows.

- Temporal: all readings are related to timestamps that are important to form historical trajectories, which determine temporal-oriented data lineages for tracking and tracing RFID objects. When RFID readings happen, RFID objects are associated with different timestamps in different positions, and different RFID objects are associated with the same timestamps in the same position (e.g., containment relationship).
- Position: RFID readings form a series of different related position data as a data lineage for a RFID object. Position information of RFID objects determines historical trajectories according to their time sequences. Containment information determines hierarchical relationships among RFID objects and implies important logic information. For example, a pallet is loaded with cases, and a truck leaving the warehouse implies that all its contained objects leave the warehouse as well. In addition, the relationship may be changed in the shipping processing. For example, a pallet is unloaded, and some objects contained in the pallet are removed to other pallets. This means that the containment relationship is changed among the pallets and the objects.
- Granularity: for most RFID applications in supply chains, users commonly focus on rough timestamps, position, and route. For example, a customer books a batch of phones at 9:00 am and wants to know these phones' position at 9:00 pm. The phones are from manufacturer *M* to dealer *D*, and they are in a certain warehouse *W* about 8:00 pm and in a certain transshipment point *T* about 8:50 pm according to its position data. If phones do not have position data at 9:00 pm, we may give a rough position covering the warehouse and the transshipment point and a rough route from *M* to *D*. The information is enough for the customer though given position and route are imprecise. It is not necessary for the customer to obtain detailed information (e.g., the phones are in *W* at 9:00 00:000 pm and they are from *W* to *T*).
- Uncertainty: since RFID devices are the intrinsic sensitivity to environmental factors, uncertainties of RFID data may be raised by RFID readers or environmental factors. We summarize several uncertainty features of RFID data as follows.
  - Uncertainty of positions: some RFID readers perform normally in certain positions but may fail in a particular position (e.g., places surrounded by water or metal). This means that environmental problems might exist in that position, which needs to be resolved for correct readings.
  - Uncertainty of RFID readers: most RFID readers can correctly perform readings, but some RFID readers cannot read normally due to, for example, malfunction. This means that the abnormal RFID readers need to be repaired.

### 3.3. Entities in RFID Applications

We identify several fundamental entities that are directly used in RFID applications as follows [3, 4].

- Object: objects are read to express their situations in the supply chain at a series of timestamps by different RFID readers with different positions. EPC-tagged objects with unique code may be items, carriers, cases, pallets, and so on. An object may contain another object. For example, a pallet may have a number of cases.
- Reader: EPC-tagged readers with unique code may be fixed or moveable. Here we design readers to process businesses during a special period. Fox example, a reader may read unloading operations for RFID objects in the morning and may be changed to read packing operations for RFID objects in the afternoon.
- Sensor: sensors measure some parameters (e.g., temperature or position) of RFID objects and write measurements to their attached RFID tags. Here we design sensors that may be attached on RFID objects or positions with EPC tags. For example, GPS sensors are attached on EPC-tagged trucks for measuring their current positions.
- Position: positions of EPC-tagged objects may be physical (e.g., point measured by GPS) or symbolic position such as EPC-tagged warehouses, retail markets, and retail stores. Similar to objects, positions also have containment relationships. For example, a shopping mall may contain a retail store.

There are other entities such as owners of positions and readers, properties of objects, and businesses of readers.

### 3.4. RFID Data

Entity relationships of RFID applications are similar to the traditional ER model, which generates relational table according to correspondence between entities. However, RFID data are massive, so inconsistent data that violate integrity constraints need to be stored in RFID databases. This raises an issue that relational tables cannot completely employ the traditional method to create data model. In supply chains, some data exist when RFID applications are deployed. These data are relatively static. However, positions of RFID objects and readers and their containment relationships are dynamically changed. In the following, we summarize three types of data.

- Deployment data: the deployment data are relatively stable and rarely changed. Such data are certain and consistent. For example, the position of a fixed RFID reader is often unchanged.
- Reading data: the reading data are directly obtained while RFID readers read EPC tags or sensors sense parameters. The data may be inconsistent, redundant, and incomplete.
- Outline data: the outline data are processed into some relational tables according to reading data. Since users focus on positions and routes of objects at a certain time, these data are very helpful for tracking and tracing objects.

We summarize uncertain RFID data as the following different groups.

- Missing data: RFID readings are noisy in actual situations. This may be due to many reasons such as bad reading angles, blocked signals, or signal dead zones. This results in weak or no signal readings that are eliminated by the physical cleaning. Specially, RFID readers might not capture containment relationships of interobjects. Missing readings result in lack of objects' certain positions. Thus, position information need to be automatically inferred by related algorithms according to some conditions. Generally, missing data are the main source in the uncertainty of RFID data. For example, the object *B* is not read at the warehouse by a reader (i.e., missing reading), so the position of *B* needs to be inferred by related algorithms.
- Inconsistent data: Different RFID readers might capture different readings for the same RFID objects. Data cleaning techniques can clean inconsistent data, but these techniques are semiautomatic and cannot satisfy requirements for processing massive RFID data. Hence, inconsistent data need to be stored in RFID databases. Inconsistent data also violate integrity constraints such as functional dependencies (FDs). For example, the object *C* is read by two different readers with different positions; the object position also is regarded as inconsistent position. It is difficult to identify which position contains the object *C*. Thus, data inconsistencies are inevitably stored in RFID databases.
- Ghost data: radio frequencies in reading areas might cause RFID readers to obtain inexistent RFID objects. In general, ghost readings rarely appear in multipositions in supply chains. For example, the object *F* is inexistent, but it is read as a ghost reading. Since the object *F* is rarely read at the other positions, the data of the object *F* should be temporarily stored in the RFID database. Once ghost data are distinguished from other types of uncertain data, they will be cleaned.
- Redundant data: wired RFID readers frequently read in powered environments, and thus they can create massive data. However, the stored data may include significant amounts of redundant information such as unchanged object positions. The redundant data can be filtered by identifying their unique identifiers. For example, the object *D* is repeatedly read as a redundant reading, which generates two copies of data. Redundant data need to be cleaned by identifying their unique identifier. If positions of RFID objects are unchanged, reading timestamps will be stored for inferring its position when they are lost at the closest time.

For incomplete data, we distinguish two types of data.

- Incomplete data (fake): fake products produced by an abnormal manufacturer might enter in a certain node of supply chains and mix with normal products. In general, data for fake products only exist in the midstream and the downstream of the supply chains. For example, the object *G* is produced as a fake by an abnormal manufacturer. It is added into the midstream or the downstream of the supper chain. This is similar to ghost reading, but the objects indeed exist. Therefore, the objects should not be cleaned.
- Incomplete data (stolen object): EPC-tagged objects might be stolen. The data on these stolen objects would normally not appear in the downstream of the supply chains. For example, the object *D* is stolen and does not appear in certain nodes of the supply chain (e.g., it skips wholesaler and appears directly in retailers).

We represent raw RFID data as a tuple for supporting data cleaning. Our model incorporates RFID data's application information, including tag ID, time, and position. These original data tuples form a spatial-temporal data streams *DS*.

**Definition 1 (data streams)**

An observed raw RFID reading is defined as a tuple *ds*_*i*_ = (*d* ^*i*^, *t* ^*i*^, *l* ^*i*^), where *d* ^*i*^, *t* ^*i*^, *l* ^*i*^ denotes the “tag ID”, “timestamp”, and “position.” And an RFID data stream is a spatial-temporal sequence of tuples *DS* = 〈*ds*_1_, *ds*_2_, *ds*_*k*_〉, where each tuple *ds*_*i*_ ∈ *DS* is represented as *ds*_*i*_ = (*d* ^*i*^, *t* ^*i*^, *l* ^*i*^), where *d* ^*i*^, *t* ^*i*^, *l* ^*i*^ denotes the “tag ID”, “timestamp”, and “position.”

For instance, one instance of a data tuple (EPC0001, 2:05 pm, Sydney-D) indicates that, at 2:05 pm, the object with ID of EPC0001 was located at Sydney Distributor (i.e., Sydney-D). At 3:05 pm, however, this object may be at Melbourne Wholesaler, which could be represented as another tuple (EPC0001, 3:05 pm, Melbourne-W).

**Definition 2 (problem statement)**

Given a stream of raw RFID readings *DS* = 〈*ds*_1_, *ds*_2_, *ds*_*k*_〉, which could be noisy, we aim to derive a clan data steam to support path-oriented queries.

### 3.5. A System View of the Preprocessing Framework

**Figure 2:**
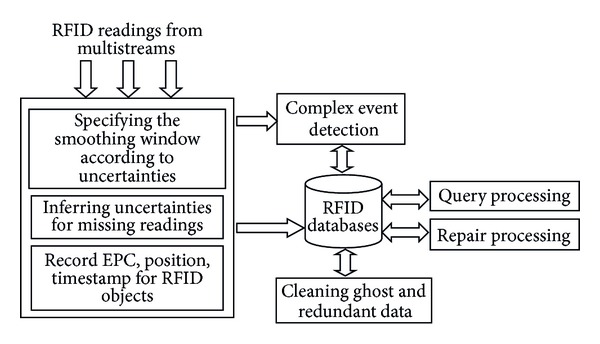
A system view of our proposed framework to process RFID data.

In this section, we briefly present our preprocessing framework (see Figure 2) and illustrate the repairing mechanism to the preprocessed data that are stored in several phases.

In Figure 2, RFID readers read EPC tags from multistreams. A smoothing window is specified according to the rate of uncertainties by analysing existent readings. Next, uncertainties of missing readings are inferred by related algorithms. Redundant readings need to be temporarily stored in databases and may be helpful to infer missing readings, which usually are the latest redundant readings. Since distinguishing ghost and inconsistent readings depends on further readings, these readings record EPC, position, and timestamp of RFID objects. The above reading information is stored in RFID databases. Moreover, RFID applications also detect and process complex events, which consist of single events with logic relations.

Ghost data are distinguished from uncertain data such as incomplete (fake or stolen object) and missing data. Ghost data and redundant data will be cleaned. Repair processing will modify inconsistent data by aggregating interval time, position, and path. Query processing focuses on tracking and tracing according to data lineage. RFID data warehouse stores historical data, which will be mined for obtaining valuable information.

Here we consider the performance of Online Transaction Processing (OLTP) about RFID databases. Cleaning data may be executed offline (e.g., night) by employing suitable programs (e.g., database triggers), which does not affect the performance of OLTP. Moreover, if frequent modifications generate large amount of database fragments, the fragments may be arranged during an interval time (e.g., per 2 weeks).

## 4. The Preprocessing Strategies for Uncertain RFID Data

### 4.1. Different Smoothing Windows for Uncertain Reading

A smoothing window significantly affects the rate of redundant, inconsistent, missing, and ghost data. The challenge is that there is a trade-off in setting the window size for tracking uncertain EPC-tagged objects. On the one hand, if we choose a bigger window size, we are able to accurately capture more information of EPC-tagged objects. However, this might raise more ghost, redundant, and inconsistent readings. On the other hand, if we choose a smaller window size, we are able to correct more uncertain readings due to tag jamming or blocking. However, this might raise more missing readings because raw data readings can only be irregularly read by readers in the presence period of EPC-tagged objects. Since “smoothing filter” like SMURF [23] is not effective to determine a variable window for all possible RFID data streams, we develop an adjustable strategy to process uncertain readings from different window sizes over RFID data streams.

**Figure 3:**
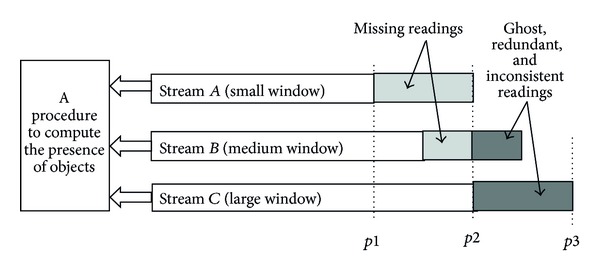
Using three different smoothing windows for reading RFID objects.

To illustrate the idea of the adjustable strategy on the data streams, we show a simplified diagram in Figure 3. Stream *A* (small window) should have a large window whenever missing readings frequently happens (e.g., from point *p*1 to point *p*2). However, stream *C* (large window) should have a smaller window whenever ghost, redundant, and inconsistent readings frequently happen (e.g., from the point *p*2to the point *p*3). Stream *B* (medium window) might be “neutral” such that it tends to balance the two types of readings (e.g., the point *p*2).

According to the rate of missing data from a certain data stream with small window (e.g., stream *A*), the small window may be increased to a suitable size level. Similarly, the window of stream *C* may be decreased to a suitable size level according to the rate of ghost, redundant, and inconsistent data from a certain data stream with large window. The strategy uses more different sizes of windows, which can adjust the rate of uncertain data over multistreams obtained by different antennae configurations of RFID readers. Here we employ a formula to adjust the size of smoothing windows as the following:

(1) $$\begin{array}{c} sw=adj*\left(m*\left(acra-ra1\right)-n*\left(acra-ra2\right)\right), \end{array}$$

where *sw* is the size of smoothing windows for reading EPC tags at the current position; *acra* is the acceptable rate of uncertain data; *ra*1 is the rate of the latest referable ghost and redundant data at the current position; *ra*2 is the rate of the latest inconsistent and missing data at the current position; *adj* is the adjustable interval time; *m* and *n* are parameters to adjust *ra*1 and *ra*2 for the size of smoothing windows, respectively.

### 4.2. Different Rules for Inferring Uncertain and Incomplete Data

**Figure 4:**
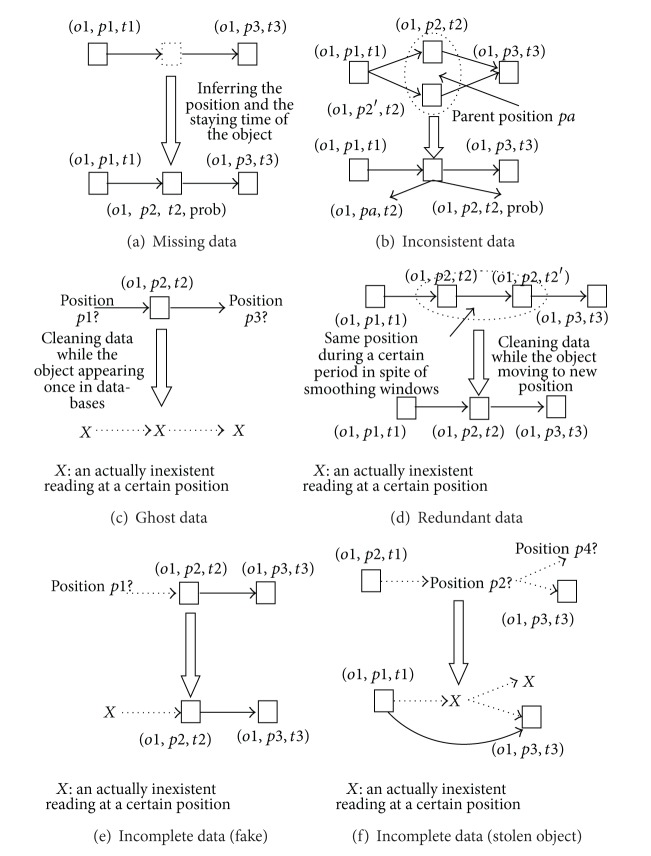
Inferring uncertain and incomplete data.

To further process uncertain data, the challenge is how to develop inference rules to process the data. We highlight interesting scenarios using several classes in Figure 4, where the pattern* EPC, position, t*_*in*_, and*t*_*out*_ generated by* EPC, position,* and* timestamp* denotes the fact that an EPC-tagged object* EPC* is detected within a time interval “[*t*_*in*_, *t*_*out*_]” measured in seconds at spot position.

Our inferring rule consists of three parts:* pattern*,* condition, *and* action*. First, a pattern represents an ordered data list in data stream in input. For example, the two consecutive readings *A* and *B* in a data stream are represented as (*A*, *B*), which means *A* and *B* are adjacent in their position relations within the data stream. Second, a condition specifies the existential semantics of the patterns specified. For example, if the two duplicate readings *A* and *B* in a data stream are coming, it is true that *A*'s position is the same as that of *B*. Finally, action part of the inferring rule indicates the designed operations to be performed when the condition is satisfied. For example, if the data stream *B* is a duplicate data with respect to* A*, then a Delete operation will be applied to remove the data stream *B*.

**Definition 3 (inferring rule)**

A inferring rule is defined as a Pattern-Condition-Action (PCA) rule:

INFERRING RULE 〈rule ID〉

PATTERN 〈pattern〉 CONDITION 〈condition〉

ACTION 〈action〉

If some patterns appear in data streams and conditions are satisfied, then an action is performed to add or drop data.

*Missing Data.* Inferring missing data deals with the following scenario that is shown in Figure 4(a). When* o1* goes through a sequence of readers (or positions)* p1*,* p2,* and* p3*,* p2* fails to detect* o1*. To infer the position, we may infer the position of* o1* according to the relationships of the readers or the containment information of objects. For example, if a reader is at* p1 *and a reader is at* p3*, the* o1 *going from* p1* to* p3 *must pass* p2*. In most situations, items are contained in boxes and boxes are contained in pallets, and they are moved together. Thus it is possible to infer missing data in certain positions if other normal readings in the same containment are recorded. In general, since objects are tagged in the manufactures, missing readings do not happen on product lines. EPC-tagged objects should appear at the positions of manufactures in databases. Even if missing readings happen, they may appear at the warehouses of manufactures.

In addition, another rule denoted as particle filtering is to evaluate the sample distribution of the responding readings and to infer the lower level position information according to obvious readers' positions. The rule maintains the sample list under the hidden situation, weighs the samples as approximate position, and assigns their probabilities according to reading values.

*Inconsistent Data.* Modifying inconsistent positiondeals with a usual scenario which is shown in Figure 4(b):* o1 *goes from* p1 *to* p3* passing through* p2* but it is also read by a near reader placed in another position *p*2′. To obtain the certain path for inconsistent data, we employ two rules. The first rule is similar to inferring missing data, which refers to the relationships of the readers or the position information of related objects. Another rule will store the parent position* pa* of* p2* and *p*2′ if* o1* cannot refer to related information. For example, a reader* r1* and another reader* r2*, respectively, locate at position* p2* and *p*2′ in the warehouse* w1*, and* r1* and* r2 *all read* o1*. However,* o1 *lack the position information of other related objects and close position information of* p2* and *p*2′ during the latest time interval. As a result,* o1* should be at* pa* which contains* p2 *and *p*2′, and* o1* should be stored at* o1, pa, t2*.

*Ghost Data.* Cleaning ghost data deals with a casual scenario that is shown in Figure 4(c): due to an environmental reason, an inexistent object* o1 *is read by a reader placed in the position* p2*. In fact,* o1* should not be read at the other positions as *X* → *X* → *X* (*X* is an inexistent reading for an inexistent object at a certain position *X*). However, if an object is stolen immediately after it leaves the product line, this might confuse the ghost data and stolen data because they all only appear once in the database, so we distinguish the situation from ghost data (see the next section). This can avoid that an actual object is mistaken for an inexistent one. Also, ghost data should be periodically cleaned.

*Redundant Data.* Cleaning redundant data deals with a usual scenario shown in Figure 4(d): an object may be frequently read within several smoothing windows, but the object does not change its position during the period. For example,* o1 *is read twice within different smoothing windows from the timestamp* t2* to the timestamp *t*2′. However,* o1* may be missed, so inferring the missed positions of* o1* needs these redundant readings at* p2*. Though redundant data are read within different smoothing windows, we will only store the check-in timestamp and the check-out one at the unchanged position. In general, we may periodically clean the redundant data.

*Incomplete Data *(fake). A case of incomplete data is shown in Figure 4(e): fakes enter and mix with normal EPC-tagged objects in the midstream and the downstream of supply chain such as wholesalers or dealers. Specially, fakes do not generally appear at manufactures of supply chain. For example, the fake* o1* appears at* p2* and* p3, *and* o1* does not appear at the position* p1*, so its path may be described as *X* → *p*2 → *p*3. Since fakes actually exist in the supply chain, we propose that fakes' data should not be cleaned if they appear twice or more. Specially, we highlight a type of fake with cloned EPCs, which also appear at the product line. There are two special situations: (a) fakes and their related authentic products might simultaneously appear at the supply chain; (b) fakes appear in the supply chain, but their related authentic products do not appear in the supply chain because they are sold or stolen. As a result, we need to distinguish these two situations.

*Incomplete Data (Stolen Object).* Another case of incomplete data is shown in Figure 4(f): objects are stolen at a certain position, so they would not be read at the position. The objects may enter the same supply chain again by other paths. Also, the objects may be lost forever from the supply chain. For example,* o1* appears at the position* p1*, but* o1 *is stolen at the position* p2*. In addition,* o1 *was not read at* p2*, and* o1* may be lost as *p*1 → *X* → *X* or again appear at the position* p3* as *p*1 → *X* → *p*3 (e.g., stealers transfer the objects to the downstream of supply chain). It is similar to fakes, so we also do not clean stolen objects' data.

### 4.3. Distinguishing Ghost, Missing, and Incomplete Data

**Figure 5:**
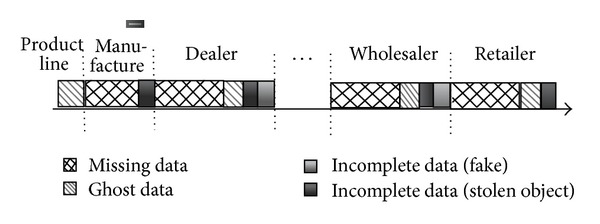
Appearance positions for ghost, missing, and incomplete data in supply chains.

There are different rules to process ghost, missing, and incomplete data. However, the three types of data all lack some information in several nodes of supply chains. It is difficult to immediately distinguish them during the reading periods. For example,* o1 *appears at the position* p1*, but* o1* may be a ghost reading or an incomplete reading (e.g., fake).  Figure 5 shows appearance positions for ghost, missing, and incomplete data in the supply chain.

Since objects are tagged one by one on product lines of manufactures, missing data are nearly impossible. As a result, Missing data may happen at all positions except product lines. However, EPC-tagged objects are transferred to other positions, so missing data are frequent.

In general, fakes only enter the midstream and the downstream of supply chain, and they cannot enter the product line. If fakes mix normal objects together except in the product line and retailers, they should be tagged into the supply chain. Specially, fakes should not be tagged into retailers in the supply chain because fakes may be straightly sold to customers.

Stealing EPC-tagged objects is rare on the product line. Even if objects are stolen, this must happen before tagging. Stealing objects might appear at the other positions of the supply chain. Stolen objects might disappear or enter the supply chain again.

Ghost data may happen at all positions. Specially, there only exist ghost data on product lines of manufactures in the supply chain.

**Figure 6:**
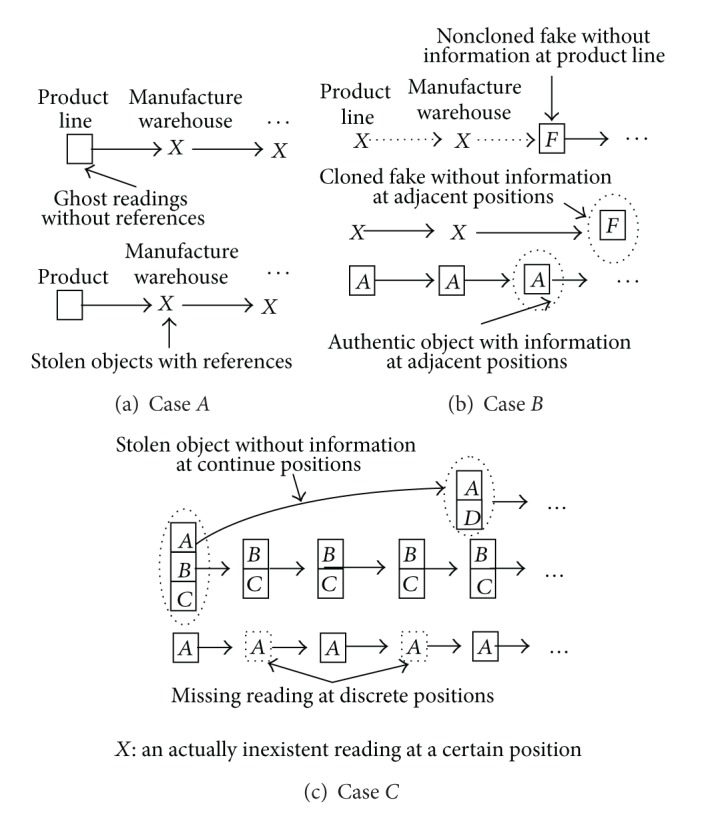
Three special cases for distinguishing ghost, missing, and incomplete data in supply chains.

Moreover, we highlight three special cases for distinguishing ghost, missing, and incomplete data in Figure 6 as follows.

- Case *A* shows that a reading only appears at the product line. This case might be a ghost reading at the product line or an object with a normal reading at the product line which is stolen before it enters the manufacture warehouse. Since EPC-tagged objects should be written into some product references, we may distinguish them by product references: ghost readings without references and the stolen objects with references.
- Case *B* shows that noncloned fakes without information appear at the product line and cloned fakes without information appear at the adjacent positions. Since EPC-tagged objects continually move, they have continuous position information. However, cloned fakes suddenly appear in the supply chain, they lack adjacent position information.
- Case *C* shows that an object appears more than once, but it does not appear at the certain position(s). This may happen in two situations: (a) the object is missed at the certain position(s) or (b) it enters the supply chain again after it is stolen. In this case, a stolen object might disappear at some adjacent positions and a missing object might disappear at some nonadjacent positions, and it is possible that its group is changed to the other one (e.g., the stolen object *A*, the normal object *B*, and *C* are in the same group at first, but *A* and *D* are in the same group later), so we may distinguish the object and infer whether it is stolen according to the continuity of the object and grouping.

Since distinguishing ghost, missing, and incomplete data will significantly increase the system workload, we propose that the work is executed when the RFID applications are in free time. This does not affect the workload of OLTP.

## 5. Modeling RFID Data 

### 5.1. Data Model

Our model will focus on a comprehensive expression for processing uncertain RFID data. There are several mapping types from the data model to relational tables according to common ER mapping rules. Entities are mapped directly as entity tables. A relationship is mapped as a table consisting of keys from among entities with the many-to-many relationship. A relationship from both entities without the many-to-many relationship is not mapped as a new table. Key attributes of master tables should be added into child tables as foreign key attributes.

**Figure 7:**
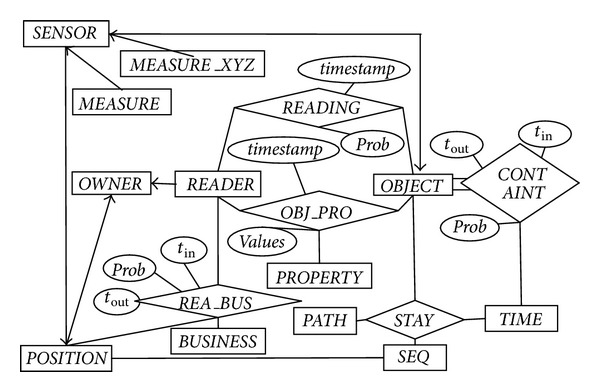
Data model.

In Figure 7, we present a model of RFID data, which may be extended by RFID applications. RFID readers read EPC-tagged objects and store raw data as* EPC, reader_ip,* and* timestamp*, where* EPC* is a unique ID of a tag and* reader_ip* is a unique RFID reader ID at the position of* EPC *and* timestamp* is the time instant of reading* EPC*. We obtain the position of* EPC* by connecting* REA_BUS*, which expresses positions and businesses (e.g., unloading situation) of readers. We further transfer raw data into the* STAY *table for expressing an RFID tag moving through a position within a time interval.

### 5.2. Tables for Deployment Data

Several fundamental entities are* READER*,* OBJECT*,* SENSOR*,* TIME*,* POSITION*,* BUSINESS*,* OWNER*, and* PROPERTY*.

The* READER *(*reader_id, name, *and* owner_id*) table stores information of readers about EPC, name, and owner ID. A reader belongs to a certain owner, so the* READER* table may connect to the* OWNER* table by the* owner_id *attribute.

The* OBJECT* (*EPC, description, container_id, *and* item_id*) table stores information of objects about EPC, container type, and item type. Also, the* OBJECT* table may connect to the* CONTAINERTYPE* and* ITEMTYPE*. The table mainly expresses objects' information, but it does not express the containment relationship histories, which are expressed by the table* CONTAIN* (see next section).

The* SENSOR* (*sensor_id, attachment, name, type,* and* measurement unit*) table stores information of sensors about sensor ID, attachment with sensor, name, type, and measurement unit. Here we consider two situations: (a) a sensor may be attached to an object, and it may be moved to other places with the object; (b) a sensor may be fixed at a certain position, and it checks parameters at the position; it may be moved to other places to check parameters, but it is not attached to an object. In addition, a sensor's attachment may be a different scope for objects and positions. For example, a sensor may be attached to a truck or a pallet, or it may be attached at a retailer store or a market too.

The* POSITION* (*position_id, name, parent_id, x, y, z, owner, *and* prob*) table stores information of positions about ID, name, parent ID of position, coordinates, and owner. In the table, we set that the zone of the attribute* parent_id* (e.g., market* A*) includes the zone of the attribute* postion_id *(e.g., retailer store* A*). In addition, readers may be movable and positions may be continuous. In addition, a position corresponds to a concrete coordinate; we can obtain the concrete position according to the concrete coordinates. An example of this situation is continuous cargo management. In order to consider the situation, special attributes as continuous coordinates should be added to the table, which might be identified by a position sensor (e.g., GPS). Similarly, a position belongs to an owner. Since there is intrinsic sensitivity to environmental factors in some positions, we will add the* prob* attribute into the* POSITION* table for expressing the uncertainties of environmental factors. The* prob* values will be computed by related algorithms according to the rate of uncertain data.

The table* OWNER* (*id, name*) stores owner information of readers and positions such as wholesalers or suppliers.

The table* PROPERTY* (*id, name*) stores property information of objects such as weight or color.

The table* BUSINESS* (*id, name*) stores business types such as packing or unloading. Different readers may process different businesses. For example, some readers often read packing data, and other readers often read unloading data. But the function of readers that reads the packing may be changed to read the unloading transactions. Similarly, businesses of objects are obtained at a certain time according to* OBJECT, BUSINESS* and* OBJ_BUS*.

The table* TIME* (*t*_*in*_, *t*_*out*_) stores rough or precise time intervals. For example, an object is uploaded at a timestamp, and its timestamp may not be different with other objects' timestamps, but their timestamps are continuous within the same time interval. Specially, the table is related to* SEQ* and* CONTAIN*, and *t*_*in*_ and *t*_*out*_ are assigned suitable values according to concrete situations in RFID applications.

### 5.3. Tables for Reading Data

Tables for reading data include* READING* generated from* OBJECT *and* READER, OBJ_PRO* generated from* OBJECT, PROPERTY* and* READER, MEASURE_XYZ, *and* MEASURE *generated from* SENSOR*.

The table* READING* (*reader_id, EPC, timestamp, *and* prob*) stores raw reading data including reader's EPC, tag's EPC, the reading timestamp, and the probability. Uncertain data are mainly present as missing readings, which are inferred into the table by related algorithms. Specially, noncloned fakes, cloned fakes, stolen objects, and normal objects are stored into* prob*, their values are 2, 1, 0, and NULL, respectively. Also, probabilities of missing readings are in the interval (0, 1).

*MEASURE_XYZ* (*sensor_id, timestamp, x, y, *and* z*) expresses that a sensor obtains concrete coordinates at a certain time. The coordinate may be computed as a position according to the* POSITION* table's coordinates. Here we consider that the coordinate of the* MEASURE_XYZ* table is approximate to the coordinate of the* POSITION* table. The coordinate of the* MEASURE_XYZ* table is not consistent with the coordinate of the* POSITION* table when the sensor is fixed, and the coordinate of the* POSITION* table should be correct.

*MEASUREMENT* (*sensor_id, timestamp, *and* values*) stores measurement information of sensors at a certain time. Since sensors are often attached at positions or objects' tags, measurements are rarely lost.

*OBJ_PRO* (*EPC, property_id, reader_id, timestamp,* and* values*) stores property information of objects at a certain time. Properties of objects may be written by a reader. The* OBJ_PRO* table mainly expresses that readers write data into objects and it is different from the* READING* table, which is read by readers. As a result, a reader that reads an object may be a different one from a writing reader of these data.

### 5.4. Tables for Outline Data

Outline data are very important for users to track and trace positions of objects. Tables for Outline data include* CONTAIN *generated from* OBJECT* and itself,* STAY, SEQ* generated from* PATH, OBJECT* and* POSITION*;* REA_BUS *generated from* READER* and* BUSINESS*, and OBJ_PRO generated from* OBJECT *and* READER*. We add an interval [*t*_*in*_, *t*_*out*_] to express the period for outline data or add a timestamp to express the occurring time for reading data. In addition, in order to express uncertainties of RFID readers, positions of objects, and containment relationships, we add* prob *attribute into* REARDING, REA_BUS, POSITION*, and* CONTAIN*, respectively.

The table* REA_BUS* (*reader_id, t*_*in*_, *t*_*out*_ *, business, position,* and* prob*) stores businesses of readers during certain time including reader EPC, start time, end time, business, and reader uncertainty. Since readers often miss or misread data, we add a special the attribute* prob* for expressing probabilities of readers. The probability needs to be computed by related algorithms. Specially, if reader's probability is null, it is a reliable reader.

The table* CONTAIN* (*EPC, parent_EPC, t*_*in*_, *t*_*out*_, and* prob*) stores containment relationships between objects, including object EPC, parent object EPC, start time, end time, and probability. Though containment levels may be more than 2, this implies that self-join queries are a big overload. But most containment levels are 2 and these queries are rare. We do not consider the multilevel containment relationship in order to lower the overload of self-join queries.

In general, uncertain data are missing, so positions of objects should be inferred by related algorithms. We consider that the* READING* table stores the data readers read, and the inferred data would be stored in the* CONTAIN* tables. Inferred data may not be read by readers. In other words,* EPC* attribute values of the* CONTAIN* table may not be in* EPC* attribute values of the* READING* table.

The table* PATH *(*path_id, parent, *and* weight*) stores path sequences as directed graph (see the latter section). The table includes position ID, its parent position, and* weight* from* parent* to* path_id*. This can distinguish uncertain data according to path sequences.

The table* SEQ* (*sqid, postion_id, t*_*in*_, and *t*_*out*_) stores positions during a series of periods. This can compress the data volume for efficiently executing queries. In Table 1, the sequence* sq1* indicates that the reading happened at the position* d1* from the timestamp *t*1′ to *t*3′.

The table* STAY* (*EPC, sqid*) stores sequent paths of objects. In Table 2, the object *a* has a sequent path *sq*1 → *sq*2, and the object *b* has a sequent path *sq*1 → *sq*3. This indicates that *a* and *b* have a partial same path *sq*1.

In general, users do not concern about positions of sensors at a certain time, so it is not necessary to generate a table for easily querying positions of sensors. We may query them by connecting related tables such as* READER, READING, OBJECT, SEQ*, and* STAY*.

Different types of data are stored in different tables shown in Table 3.* OBJECT* is the original information about EPC-tagged objects; it should not store uncertain data.* CONTAIN, SEQ*, and* STAY* are the outline data, and these tables only store inferred data for missing readings. Since* READING* contains the original data of all readings, it should not store inferred data for missing readings.

In Table 4, we focus on tables in the different stages of the supply chain such as deployment, production, warehouse, transfer, and retail.* OBJECT* and* OBJ_PRO* store information of objects at the production stage.* POSITION, READER, SENSOR, PROPERTY, OWNER, TIME*, and* BUSINESS* are relatively static; their information is generally stored at the deployment stage. Since readers' business may be changed,* REA_BUS* may be modified at every stage except transfer.* READING, MEASURE, CONTAIN, SEQ*, and* STAY* store outline data, information of readings, and measurements read by readers or sensors at the production, warehouse, and retail stages.* MEASURE_XYZ* stores coordinate information at the transfer stage.

## 6. Path Coding Scheme

### 6.1. Aggregations for Object Paths

The reading data in each position are sent to the database. Then, the data are generally generated into stay records in the form of (*EPC, position, t*_*in*_, and *t*_*out*_).* READING* may have many redundant data, so the data volume of* READING* needs to be compressed into stay records. This is helpful to efficiently execute users' path queries. We may also represent how long an object* EPC* stays at position from *t*_*in*_ to *t*_*out*_ in stay records with historical paths.

According to features of the supply chain, EPC-tagged objects may be transferred by grouping or single. Gonzalez et al. [25, 26] only focus on group of objects and use compression to preserve object transition relationships as a compact representation of RFID data sets. Lee and Chung [27] only focus on single objects and propose a movement path of an EPC-tagged object, which is coded as a series of unique prime number pairs (*Pin, Pout*). Employing* Unique Factorization Theorem *can code* Pin* as positions of readers, and employing* Chinese Remainder Theorem *can code* Pout* as the order of positions. However, the work [27] cannot code long and cyclic paths, which may incur the overflow of the prime numbers. For example, defective objects transferred from manufactures to retailers need to be retreated from retailers to manufactures. The path is “*P*1 → *P*2 → ⋯→*P*16,” and its path length is 2 × 3 × 5 × 7 × 11 × 13 × 17 × 19 × 23 × 29 × 31 × 37 × 41 × 43 × 47 × 53, which is greater than 2^64^−1 (64 bits). To partial computing (32 bits), the method only supports the product of the first 8 prime numbers as 2 × 3 × 5 × 7 × 11 × 13 × 17 × 19. In addition, since an object may have the same position with different time intervals,* Chinese Remainder Theorem* is not applicable to express cyclic paths. Ng [31] first partitions the whole set of positions into different clusters having roughly the same number of positions. Using finite continued fraction (rational numbers) represents a cluster code together with its respective* Pin, Pout*, which can be straightforwardly extended into more than one level within each cluster. This can resolve the limitation of 8 prime numbers for coding a path in a cluster. Also, Ng [31] employs* Euler's formula* to generate prime numbers for the same positions in cyclic paths. However, rational numbers notably increase the volume of data and lead to inefficient queries.

**Figure 8:**
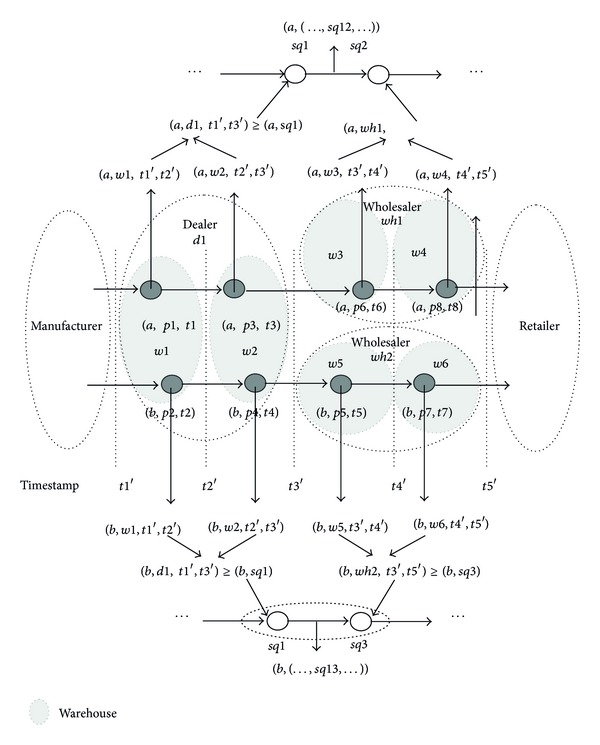
Aggregations for RFID data.

It is inefficient to support path queries, which need to perform multiself-joining of* STAY* involving many related positions. Thus, we will extend existing coding techniques, called path (sequence), and compress data into* SEQ* (*sqid, postion_id, t*_*in*_, and *t*_*out*_) by aggregating the path sequence, the position, and the time interval (see Figure 8). In* STAY*, we employ* EPC, sqid* instead of* EPC, position, t*_*in*_, and *t*_*out*_. These aggregations resolve two problems: the cyclic path and long path, and comprehensively consider group and single objects. These significantly reduce redundant data and greatly compress the volume of data.

*Aggregating Time Intervals.* Most users do not concern about concrete timestamp of objects. For example, users may only know that the stay time of the object *a* is from *t*1′ to *t*2′. Moreover, users may enlarge the time interval which is from *t*1′ to *t*3′. As a result, though the object *a* is read at the timestamp* t1* and the object *b* is read at the timestamp* t2*, their stay time may be the same as the interval [*t*1′, *t*2′]. Users may further enlarge the time interval [*t*1′, *t*2′] to [*t*1′, *t*3′]. Though the object *a* is read at the timestamps* t1 *and* t3* andthe object *b* is read at the timestamps* t2 *and* t4*, their stay time may be the same as the interval [*t*1′, *t*3′].

*Aggregating Positions.* Similarly, most users do not concern about concrete positions of objects. For example, a customer buys a computer. It is enough to know the logistic position storing the compute rather than the concrete warehouse of the logistic position. As a result, an object at a position may be aggregated into a parent position as a single reading in spite of multireadings with different positions. In Figure 8, the object *a* is read at the position* p1* and the object *b* is read at the position* p2*, so their positions may be enlarged as the warehouse* w1*. The object *a* is read at the warehouses* w1 *and* w2*, respectively, so their positions may be further enlarged as the dealer* d1*.

*Aggregating Path Sequences.* A group of objects have the same path, so we further compress the path expression as the main path by aggregating the same child path sequences. For example, the object *b* is from the path sequence* sq1 *to* sq3*, and here we denote the path sequence* sq13* to express from* sq1 *to* sq3*. As a result, the path sequence of *b* may be denoted as *b, sq13* by aggregating* sq1* and* sq2*. In general, the aggregation is used to compress paths of group of objects. If a large number of scattered objects do not have the same path sequences, it is not necessary to aggregate path sequences.

### 6.2. Algorithms

Next, we propose several algorithms: the processing algorithm for uncertain data, the aggregating algorithm for compressing data, and the graph algorithm for building directed graph data.

Since our methods are based on the supply chain from the upstream to the downstream, we first define several key concepts for further processing.

**Definition 4**

* One describes a supply chain SC from the upstream to the downstream as a directed graph, which is denoted as three tuples *〈*V*, *E*, *W*〉*, where V is the node for the position of SC; E is the directed edge between a pair of nodes *〈*Vi*, *Vj*〉* for the object movement; W is the weight of E for the probability of the object movement. Specially, we denote the root node for the manufacture of objects as V0; every node is the child of its prior node and the parent of the following node*.

**Figure 9:**
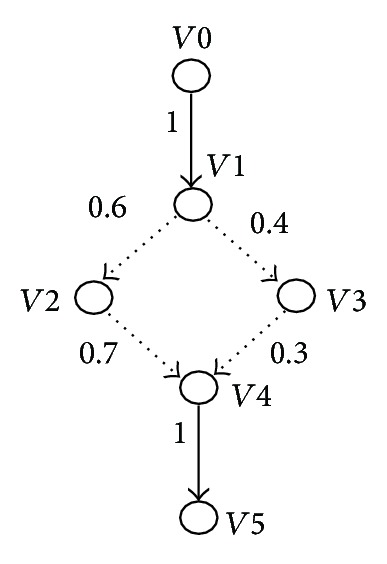
A directed graph for the object movement.

In Figure 9, we propose a directed graph from node *V*0 to node *V*5 for the object movement. The weights of the edges 〈*V*1, *V*2〉 and 〈*V*1, *V*3〉 are 0.6 and 0.4, respectively, and this denotes the probabilities from *V*1 to *V*2 and from *V*1 to *V*3 (i.e., missing or inconsistent readings might exist along the paths). Specially, the weights of the edges 〈*V*0, *V*1〉 and 〈*V*4, *V*5〉 are 1, and this indicates the probabilities from *V*0 to *V*1 and from *V*4 to *V*5 (i.e., the paths always are certain). Our algorithms deal with uncertain data based on the directed graph.

Algorithm 1 deals with uncertain data including ghost, redundant, incomplete, missing, and inconsistent data according to our analysis from Figures 4(a) to 4(f).

Algorithm 1 first calls the function Aggregate (*AT*, *AP*) (i.e., Algorithm 2) to aggregate the time interval, the position, and the path. In function* Aggregate* (*AT*, *AP*), *AT* is a parameter that indicates the level of the time interval aggregate, and *AP* is another parameter that indicates the level of the position aggregate. We do not use a parameter to aggregate paths. The reason for this is that aggregating paths may be flexible according to the group situation of EPC-tagged objects. If objects move independently, it is not necessary to aggregate their paths; if objects move in group, it is necessary to aggregate their paths according to their depths of paths. Also, the function can process the problem of fuzzy paths for missing and inconsistent data by aggregation.

Next Algorithm 1 calls the function Builddirectedgraph() (i.e., Algorithm 3) to build the directed graph and the path sequences* SEQ* according to aggregated* STAY*. Specially, positions, check-in timestamp, and check-out timestamp of the nodes in the directed graph are aggregated from* STAY*. For example, the information of nodes is significantly compressed according to multiaggregation levels. We denote the same positions as different nodes because of their different time intervals.

Algorithm 1 visits records in* READINGS* and cleans ghost and redundant data according to appearing positions of records'* EPC* and remarks data about fakes and stolen objects. The algorithm further processes missing and inconsistent data under the given threshold value condition according to weights of paths from parent to child in the directed graph.

Finally, we insert* EPC's* node into* STAY* or repair* EPC's* node in* STAY* by further aggregating higher* AT* and* AP*.

## 7. Querying RFID Data 

We note that common users want to query a rough position and a rough time interval for an object, so our framework can satisfy most users' requirements. These queries have two main types: tracking objectsand tracing the states of objects. In the next section, we will propose queries based on our framework.

### 7.1. Tracking

We track the changing history of an object's states and detect missing objects.

The following statement queries the state of the object* a *from* STAY *and* SEQ*.

 Q1. SELECT* S1.EPC, S2.postion_id, S2.t*_*in*_ *, S2.t*_*out*_
   FROM* STAY S1, SEQ S2*
   WHERE* S2.sqid* LIKE* S1.sqid%*
     AND* S1.EPC=‘a'*

The following statement queries stolen objects. Similarly, we can query fakes by employing the condition* prob*=1.

Q2. SELECT ∗ FROM* READING* WHERE* prob=0*

The following statement queriesthe time interval for object moving and return the period of the object* a* from the position* p1* to the position* p2*.

 Q3. SELECT* S2.t*_*out*_ *-S2.t*_*in*_
   FROM* STAY S1, SEQ S2*
   WHERE* S1.EPC=‘a'*
     AND* S2.position=‘p1'*
     AND* S1.sqid* LIKE* ‘p1%'*
     AND* S1.sqid* LIKE* ‘p2%'*
     AND* S2.sqid* LIKE* S1.sqid%*

The following statement returns objects in the last day at the position* p1*.

 Q4. SELECT* EPC* FROM STAY S1,SEQ S2
   WHERE* S2.sqid* LIKE* S1.sqid%*
     AND* S2.position_id=‘p1'*
     AND* S2.t*_*in*_<=SYSDATE-1

### 7.2. Tracing

Tracing objects is helpful to find resources of objects and their related objects' information in traceability RFID applications.

We query possible tainted meats, which may be tainted by known tainted meats in the same pallet. In other words, their containment relationships are the same. If the containment level is more than 3, the query may be extended as a recursive query for higher containment level. Since the periods of possible tainted meats might overlap periods of known tainted meats from start time to end time, this indicates that the tainting happens. We consider this overlap in the following query.

 Q5. SELECT* c2.epc, prob*
   FROM* CONTAIN c1, CONTAIN c2*
   WHERE* c1.parent_epc=c2.parent_epc*
     AND* c1.epc=‘TEPC'* AND
     ((*c1.t*_*in*_ *<c2.t*_*in*_AND* c1.t*_*out*_ *>c2.t*_*out*_) OR
     (*c1.t*_*in*_ *>c2.t*_*in*_AND* c1.t*_*out*_ *<c2.t*_*out*_) OR
      (*c1.t*_*in*_ *>c2.t*_*in*_AND* c1.t*_*out*_ *>c2.t*_*out*_
      AND* c1.t*_*in*_ *<c2.t*_*out*_) OR
      (*c1.t*_*in*_ *<c2.t*_*in*_AND* c1.t*_*out*_ *<c2.t*_*out*_
      and* c1.t*_*in*_ *>c2.t*_*out*_))

The following statement queries objects contained in the object* a*. The query first obtains the recursive* r *according to the object* a*, and then recursively loop according to the condition* r.EPC=c.parent_EPC *until the recursive query is terminated. The final recursive result would be returned.

 Q6. WITH RECURSIVE* r (parent_EPC, EPC*) AS
    (SELECT* parent_EPC, EPC, prob*
     FROM* CONTAIN*
     WHERE* parent_EPC=‘a'*
     UNION
     SELECT* r.parent_EPC, c.EPC,c. prob*
     FROM* a, CONTAIN c*
     WHERE* r.EPC=c.parent_EPC*)
 SELECT ∗ from* r*

The following statement tracks information of noncloned fakes by employing the condition* prob*=2. Similarly, we may employ the condition* prob*=1 to track cloned fakes.

 Q7. SELECT* S1.EPC, S2.postion_id, S2.t*_*in*_ *, S2.t*_*out*_
   FROM* STAY S1, SEQ S2*
   WHERE* S2.sqid* LIKE* S1.sqid%*
     AND* S1.EPC* in (SELECT* EPC*
               FROM* READING*
               WHERE* prob=2*)

### 7.3. Aggregation

Our framework also supports aggregation queries for RFID data. The following statement queries how many objects were in the store on 11/11/2011.

 Q8. SELECT COUNT (*S1.EPC*)
   FROM* STAY S1, SEQ S2*
   WHERE* S2.position_id=‘p1'*
     AND* S2.sqid *LIKE* S1.sqid*%
     AND* S2.t*_*in*_>=‘11/11/2011'
   AND* S2.t*_*out*_<‘11/11/2011'

The following statement queries the average time for objects moving from one position to another one.

 Q9. SELECT AVG (*S3.t*_*out*_ *– S2.t*_*in*_)
   FROM* STAY S1, SEQ S2, SEQ S3*
   WHERE* S2.sqid* LIKE* S1.sqid*%
     AND* S3.sqid* LIKE* S1.sqid*%
     AND* S2.position=‘p1'*
     AND* S3.position=‘p2'*

Based on the above framework, we can also use database triggers to specify constraints such as automatic transfer notice, low store alert, and trend analysis. The RFID queries can also be expressed with standard temporal query languages, which are yet supported by commercial RDBMS such as ORACLE and SQLSERVER.

## 8. Experiment Evaluation

### 8.1. Experimental Environment

In this section, we present the experiment evaluations for our model and algorithms. All the experiments were executed on a Core i2 2.00 GHz, with 2 Gb RAM, running Windows 7; and the code was written in MYSQL. Since there is no well-known RFID data set, we generate synthetic test data and formulate 8 queries (1 tracking query, 5 tracing queries, and 2 aggregate queries). The experiment evaluations are compared with [25–27].

We generate stay records instead of raw RFID data to reflect real environments in a real-life food distribution [32]. The objects are generated at root nodes such as import markets and agricultural input suppliers. Then, they move to the next position by grouping objects or single object in RFID applications. We consider the grouping factor to generate stay records using a directed graph for object movements and analyze how the query performance is affected by the grouping factor. Each node in the directed graph represents a set of objects in a position, and an edge represents a movement of objects between positions. In general, objects at positions near the root of the directed graph move in larger groups while objects near the leaves move in smaller groups. The size of the groups at each level of the directed graph is denoted as* G*, where* G* is the number of objects that move together in the directed graph, and* SEQ* corresponds to the object movements indicated by the edges in the directed graph. We randomly generated data according to a set of directed graphs with a given level of* G*.

Since the most of uncertain data are missing data in the whole database, we consider the effect of the rate of missing data in the whole data. We also consider the influence of the rate of redundant and ghost data in the whole data. In addition, users need to specify aggregate levels of the position and the time interval, and then the compression method automatically aggregates the path sequence based on the above specified levels. As a result, we are able to test the effect of the aggregate levels on the position and the time interval. Moreover, we also test the performance over different sizes of data. The related parameters are listed in Table 5.

### 8.2. Data Cleaning and Compression

We first evaluate the data cleaning and then study the performance of compression. We set the rates of missing data in the whole data to be *M* = (0.5, 0.25, 0.1, 0.5 and 0.025), the rates of redundant and ghost data in the whole data to be *RG* = (0, 0.333, 0.5, 0.666 and 0.75), and the number of tuples in* READING* table to be *S* = (3.8 k, 31.7 k, 135 k, 427.5 k, 975 k). The tuples are generated by 1 k, 2.5 k, 5 k, 7.5 k, 10 k objects, respectively. We will clean redundant and ghost data in* READING* table, refer missing data into* READING* table, remark incomplete data including fake and stolen data, and repair inconsistent data.

**Figure 10:**
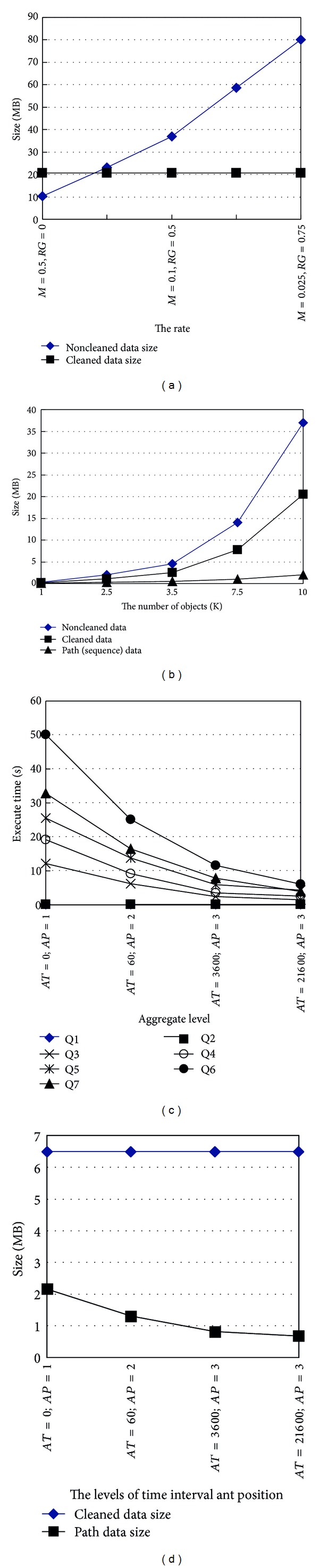
(a) Different rates of missing, redundant, and ghost data. (b) Different data sizes. (c) Different path lengths. (d) Different aggregate levels of positions and time intervals.

Since the rate of missing data is opposite to the rates of redundant and ghost data, we set several sets of rates denoted as (*M* = 0.5, *RG* = 0), (*M* = 0.25, *RG* = 0.333), (*M* = 0.1, *RG* = 0.5), (*M* = 0.05, *RG* = 0.666), and (*M* = 0.025, *RG* = 0.75). The rate of missing data declines from 0.5 to 0.025, and the rates of redundant and ghost data rise from 0 to 0.75. We generate data for 10 k objects.  Figure 10(a) shows that the sizes of noncleaned data under *S* = 10 k, *P* = 3–22, *AT* = 3600, *AP* = 3, and different *M* and *RG* linearly increase (since missing data should be inferred into* READING*, and redundant and ghost data in* READING* should be cleaned; the sizes of cleaned data under different *M* and *RG* are the same). The linear results show that the higher *RG* might significantly increase the size of* READING*, and this might increase system overloads. However, the smaller *RG* might indicate that missing data are too much to infer missing data according to related data. As a result, *RG* should be adjusted as a smaller value for efficient queries, and *M* should be adjusted as a suitable value. The two values should be adjusted according to the smoothing window.

Figure 10(b) shows a comparison for noncleaned data, cleaned data, and path data under *P* = 3–22, *AT* = 3600, *AP* = 3, *M* = 0.1, and *RG* = 0.5. In the comparison, the rate of missing data is *M* = 0.1 and the rate of redundant and ghost data is *RG* = 0.5. We generate original data into* READING* table according to the number of objects (1 k, 2.5 k, 5 k, 7.5 k, 10 k), and their path lengths are from 3 to 4, from 3 to 7, from 3 to 9, from 3 to 17, and from 3 to 22, respectively (every 500 objects with the same path length for a set objects). Since *RG* is 0.5, a long path might introduce more redundant data, and the noncleaned data size in* READING* table is obviously bigger than the cleaned data size after original data are cleaned. Moreover, we generate path sequences into* SEQ, STAY*, and* PATH.* Since* STAY* only stores a series of complete path sequence into a single tuple for a single object (objects in* STAY* can do one-to-one mapping with objects in* OBJECT*),* SEQ* only stores positions for a check-in time and a check-out time (the two times may be adjusted as a rough time interval).* PATH* only stores the common path based on the directed graph and their data size is small. Since users are interested in querying object paths, it is efficient to obtain complete object paths by querying the smaller size tables (*SEQ*,* STAY*, and* PATH*).

In general, most path lengths for object movements are from 3 to 9 (especially from 3 to 6) [32]. However, cyclic and long paths need to be considered, so we extend the maximum path length to 17 or 22 for resolving the problem in [25–27]. As a result, we set five series of path lengths: from 3 to 4 (5000 objects per path length), from 3 to 7 (2000 objects per path length), from 3 to 9 (1500 objects per path length from 3 to 8 and 100 objects for the length 9), from 3 to 17 (1300 objects per path length from 3 to 9 and 90 objects per path length from 10 to 17), and from 3 to 22 (1300 objects per path length from 3 to 9 and 90 objects from 10 to 22).  Figure 10(c) shows that the noncleaned and cleaned data sizes significantly increase when the path lengths increase under *S* = 10 k, *AT* = 3600, *AP* = 3, *M* = 0.1, and *RG* = 0.5. Also, the path data sizes slightly increase when the path lengths increase. The results show that longer paths will significantly affect original data sizes but only slightly affect path data sizes.

The above experiments do not consider aggregation factors. Since our algorithms automatically aggregate the path sequences, the levels of the positions and the time interval need to be specified by users. Here we need to consider the scenario: moving an object within a short time interval from a position to another further position is impossible (e.g., an object is transferred within 1 second from the product line to the warehouse), so a level of a position needs to correspond to a suitable a level of a time interval (e.g., the position level 2 corresponds to 1 minute, and *AT* = 60 seconds correspond to *AP* = 2).  Figure 10(d) shows that path data sizes decrease with increasing* AT* and* AP *under *S* = 10 k, *P* = 3–22, *M* = 0.1, and *RG* = 0.5 (cleaned data size is unchanged in spite of* AT* and* AP*, and these data need to be stored at their original situations for later processing for some detail applications). Decreasing path data sizes also efficiently decreases the overloads for path queries.

### 8.3. Query Processing

Since noncleaned data are unsuitable for comparisons, we employ algorithms to clean data and then aggregate data to execute the queries. We formulate 7 queries to test various features which are shown in Table 6. Q1 tests the performance of tracking query; Q2-Q3 and Q6-Q7 are tracing queries; Q4-Q5 are aggregate queries. Specially, Q6 queries incomplete data, and Q7 queries objects with a cyclic and long path.

Our method stores* EPC* and path sequences of objects in the table* STAY* (*STAY* only stores object path sequences), so Q1 and Q2 do not need to join multitables to obtain path information. Though Q3 needs to join two tables to obtain path information,* SEQ* (*SEQ* only stores the check-in and check-out time intervals of positions in spite of object EPCs) may be significantly compressed. Aggregate queries (Q4, Q5) need to join two tables to aggregate the object totals at certain positions, but quite a few similar data in two tables may be compressed by aggregating, so the compression rate may efficiently improve overloads of aggregate queries. Since fakes are related to the three tables* STAY*,* SEQ,* and* PATH* (*PATH* only stores the path graph for all objects with respect to all positions), Q6 needs to join the three tables to distinguish noncloned fakes and cloned fakes. Also, since cyclic and long paths are related to* STAY* and* SEQ*, Q7 needs to join the three tables to obtain detailed path information.

**Figure 11:**
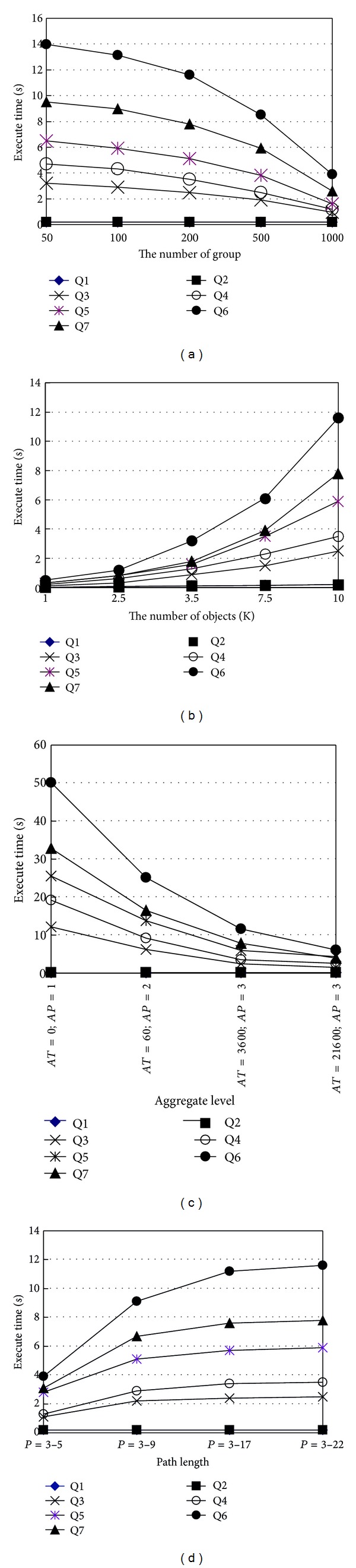
(a) Different number of groupings. (b) Different data sizes. (c) Different aggregate levels of positions and time intervals. (d) Different path lengths.

We now analyze performance of path (sequence) for different numbers of groups under *S* = 10 k, *P* = 3–22, *AT* = 3600, and *AP* = 3.  Figure 11(a) shows that path (sequence) decreases execute time when the number of group *G* increases in Q3–Q7. Because Q1 and Q2 only need to query the unique table* STAY* to obtain path information, so the execute time of Q1 and Q2 is unchanged. The execute time of Q3 decreases with *G* increasing; the reason for this is that the bigger groups may significantly compress* SEQ* for obtaining path information. Also, path (sequence) may decrease execute time when G increases in the aggregate queries (Q4, Q5). Q4 and Q5 need to join two tables (*SEQ* and* STAY*) to aggregate the object totals at certain positions, but quite a few similar data in two tables are compressed when *G* increases by aggregating *AT* = 3600, *AP* = 3, and the compression rate increases when G increases, so this may efficiently improve query overloads. Similarly, Q6 and Q7 need to join three tables (*SEQ*,* STAY* and* PATH*) to trace fakes and objects with a cyclic and long path, and the compression rate increases with *G* increasing, so this may efficiently improve query overloads.

We then evaluate Q1–Q7 under *G* = 200, *P* = 3–22, *AT* = 3600, *AP* = 3, and different data size *S* as shown in Figure 11(b). The data size significantly affect the execute time for Q3–Q7. Q3–Q7 are related to multitable operations, so the execute time of Q3–Q7 obviously increases when *S* increases. Since Q1 and Q2 only need to query the unique table* STAY*, different data sizes have influences for Q1 and Q2.

Since readings of EPC-tagged objects are within continuous time intervals without the same timestamps in RFID applications, our method proposes rough time intervals and positions to ignore concrete timestamps and positions by aggregating time intervals and positions. This may significantly improve efficiencies of storages and queries.  Figure 11(c) shows the execute time under *S* = 10 k, *G* = 200, and *P* = 3–22, and different aggregate levels. We set four sets of different aggregate levels (*AT* = 0 and *AP* = 1; *AT* = 60 and *AP* = 2; *AT* = 3600 and *AP* = 3; *AT* = 21600 and *AP* = 4) to evaluate Q1–Q7. *AT* = 0 and *AP* = 1 are without aggregations, so the situation is inefficient to execute Q3–Q7. With the aggregate level increasing, the execute time of Q3–Q7 significantly decreases.

As mentioned above, the common path lengths are from 3 to 9. We consider that most objects' path lengths are from 3 to 9 and set four sets of different path lengths (*P* = 3–5, *P* = 3–9, *P* = 3–17, and *P* = 3–22) to evaluate Q1–Q7. If the path length exceeds 9, we set 10% of the objects as the rate of objects' path lengths from 10 to 22 (relative average number per path length from 10 to 22) and set 90% as the rate of objects' path lengths from 3 to 9 (relative average number per length from 3 to 9).  Figure 11(d) shows the execute time of Q3–Q7 under *G* = 200, *S* = 10 k, *AT* = 3600, and *AP* = 3, and different path lengths. The execute time significantly increases when the path length increases. This is because shorter paths include a large number of the same path information, which is compressed by aggregating. // Longer paths include a large number of different path information, which cannot be compressed by aggregating. Since the objects with 10–22 length are rare, the trends for increasing the execute time show a slow growth.

In the above experiments, the execute time of Q6 and Q7 is slightly longer, but the two types of queries are rare, so the execute time should be acceptable.

## 9. Conclusion

One of the major challenges for RFID technology is uncertain data management. This paper proposes a framework and related algorithms to manage uncertain RFID data, which are efficiently stored and compressed into an effective store model by aggregating positions and time intervals for path-oriented object movements. Experimental evaluations show that our model and algorithms are effective and efficient in terms of the storage, and the tracking, tracing, and aggregate queries under different parameters. Our future work aims to construct the main path for object moving for further compressing data and enhancing the performance.

## Figures and Tables

**Algorithm 1:**
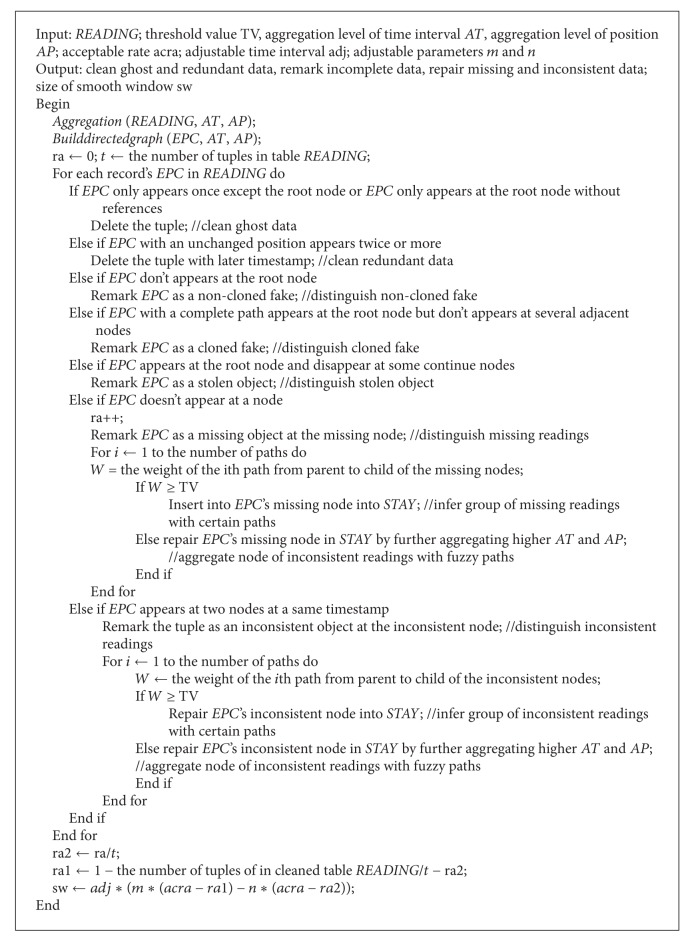
//Processing data.

**Algorithm 2:**
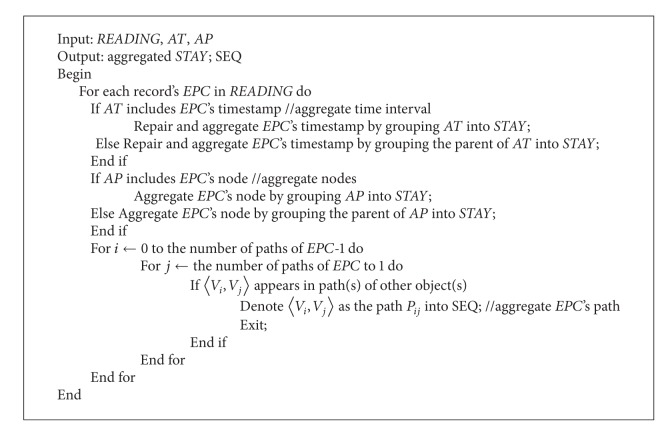
//Aggregation.

**Algorithm 3:**
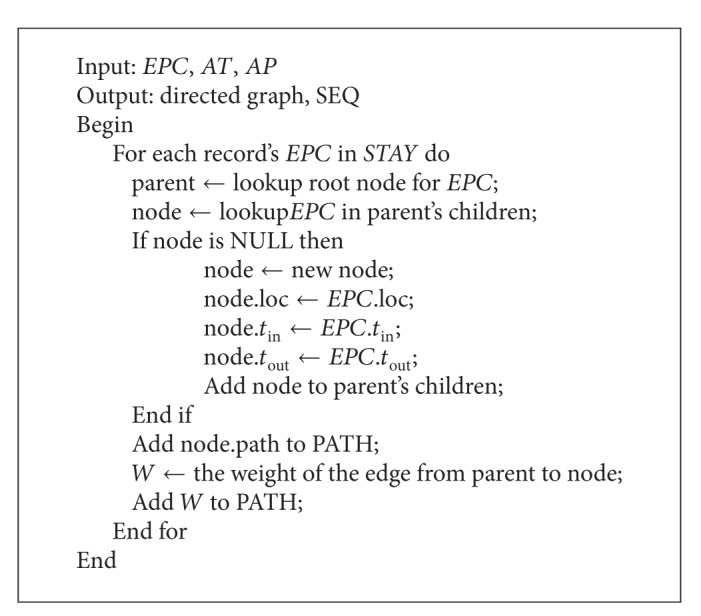
Build directed graph.

**Table 1:** Sample *SEQ*.

*sqid *	*Postion_id *	*T*_in_	*T*_out_
*sq1 *	*d1 *	*t1*′	*t3*′
*sq2 *	*wh1 *	*t3*′	*t5*′
*sq3 *	*wh2 *	*t3*′	*t5*′

**Table 2:** Sample *STAY*.

*EPC *	*sqid *
*a *	*sq1, sq2 *
*b *	*sq1, sq3 *

**Table 3:** Storing for uncertain data.

Table	Missing data	Inconsistent, redundant, and ghost data
*OBJECT *		
*CONTAIN, SEQ, STAY *	X	
*READING *	X	X

**Table 4:** Tables in the different stages of the supply chain.

Table	Deployment	Production	Warehouse	Transfer	Retail store
*OBJECT, OBJ_PRO *		X			
*POSITION, READER, SENSOR, PROPERTY, OWNER, TIME, BUSINESS *	X				
*REA_BUS *	X	X	X		X
*READING, MEASURE, SEQ, STAY, CONTAIN *		X	X		X
*MEASURE_XYZ *				X	

**Table 5:** Experimental parameters.

Parameter	Statement
*G*	The size of the groups at each level of the directed graph
*M*	The rate of missing data in the whole data
RG	The rate of redundant and ghost data in the whole data
*L*	The path length
*P*	The level of the position aggregate
*AT*	The level of the time interval aggregate
*AP*	The level of the position aggregate
*S*	The data size

**Table 6:** Test queries.

Query	Description
Q1	Tracking an object
Q2	Tracing an object without conditions
Q3	Tracing an object with conditions
Q4	Counting objects at a position without a time interval
Q5	Counting objects at a position within a time interval
Q6	Tracing fakes
Q7	Tracing objects with a cyclic and long path
